# Family Child Care Providers’ Compliance With State Physical Activity Regulations, Delaware Child Care Provider Survey, 2011

**DOI:** 10.5888/pcd10.120295

**Published:** 2013-07-11

**Authors:** Sarah Williams Leng, Laura Lessard

**Affiliations:** Author Affiliations: Sarah Williams Leng, Nemours/Alfred I. duPont Hospital for Children - Nemours Health and Prevention Services, Wilmington, Delaware.

## Abstract

**Introduction:**

Delaware is one state that has implemented comprehensive child care regulations to foster healthy dietary and physical activity behaviors of young children. This study describes the Delaware family child care environment and providers’ knowledge of and compliance with physical activity regulations. We analyzed the data to determine characteristics associated with predictors of knowledge of and compliance with these regulations.

**Methods:**

A random stratified sample of 663 licensed Delaware family child care providers was mailed a survey on family child care characteristics and providers’ awareness and practices of the child care regulations. Three logistic regression models were used to explore the association between provider characteristics and their knowledge of and compliance with the regulations.

**Results:**

Ultimately, 313 of the 663 eligible family child care providers participated in the survey (47.2% response rate). Controlling for covariates, we found that family child care providers’ education level was significantly associated with knowledge of the physical activity regulation. Another model showed that family child care providers with larger amounts of outdoor space were more likely to report compliance with the recommendation for unstructured physical activity than those without this described space (odds ratio, 2.45). A third model showed a significant association between available indoor space for all activities including running and reported greater compliance with the recommendation for structured physical activity than was reported by caregivers with less indoor space (odds ratio, 11.2).

**Conclusion:**

To provide the recommended levels of physical activity for children in child care, the available physical space environment is an important area of focus for advocates of physical activity recommendations within the family child care environment.

## Introduction

Overweight and obesity among children is an important public health focus in the United States. In 2007–2008, approximately one-quarter of children aged 2 to 5 years in the United States had a high (≥85th percentile for age) body mass index (1). Overweight and obesity are associated with a list of negative health consequences, and overweight children are more likely to become obese adults (2–4). Early childhood is an important developmental stage to foster healthy dietary and physical activity behaviors in children, and prevention efforts should begin before children enter the school system (5). In the United States, approximately 60% of children under the age of 6 are in at least 1 weekly nonparental care arrangement. Of these, 60% are in center-based care, 35% in relative care, and 22% in nonrelative care arrangements including family child care homes — child care settings where 1 or 2 adults care for a small number of children in their own home (6). Family child care settings differ from center-based settings because they operate in a private home setting and may have smaller, mixed-age groups of children in their care. Delaware has more than 1,500 licensed facilities, with 449 licensed child care centers, each providing care for 13 or more children, and 1,037 licensed family child care homes that provide care for 1 to 12 preschool-aged or younger children, depending on license type (7). Although child care center environments have been the focus of research, little is known about the family child care environment.

Improving regulations related to physical activity, nutrition, and screen time is one way to create child care environments that support healthy behaviors in children (8). Child care policy and practice on the time allotted for physical activity, which requires annual physical activity training, supportive staff behaviors, and appropriate play settings and equipment, have the potential to influence children’s physical activity levels (9–11). Most states currently do not have strong child care physical activity or nutrition regulations (12). One state that has implemented comprehensive child care regulations is Delaware. The Office of Child Care Licensing (OCCL) has regulated changes within the child care environment in the areas of physical activity that include mandated time for moderate-to-vigorous physical activity for children for 20 minutes for every 3 hours they are in care, including opportunities for both structured and unstructured physical activity (Box) (13). Unstructured physical activity refers to child-led free play and includes activities that encourage children’s individual abilities and interests and allows them to explore their environment, while structured physical activity is adult-led and includes daily planned activity that supports age-appropriate motor skill development.

**Box. Delaware Regulations and Recommendations to Family Child Caregivers for Children’s Physical Activity**

**Table:** 

**Statewide Physical Activity Regulation**
Each child should be provided with at least 20 minutes of moderate-to-vigorous physical activity (MVPA) for every 3 hours of care.
**Statewide Physical Activity Recommendations**
Children over the age of 1 year are to be provided with at least 60 total minutes of *unstructured* physical activity accumulated throughout the day.
Children over the age of 2 years are to be provided with at least 60 total minutes of *structured* physical activity accumulated throughout the day.

The research on family child care policy and practice related to physical activity is limited (10,14). This study 1) describes the Delaware family child care environment and family child care providers’ knowledge of and compliance with physical activity regulations and 2) evaluates the association between family child care characteristics and knowledge of and compliance with physical activity regulations and recommendations.

## Methods

### Study design

To obtain a representative sample within the state by geographic location, random sampling stratified by the 3 counties in Delaware (New Castle County, Kent County, and Sussex County) and the city of Wilmington (the largest city in the state) was used. Family child care homes were eligible if they were state-licensed and offered full- or part-time child care. Records for eligible licensed family child care homes were retrieved from the Delaware OCCL database of Delaware child care providers (N = 1,037) (7). From this database, 663 homes were included in the sample.

Family child care providers were mailed a cover letter informing them of the study, an informed consent form to review and sign, and a copy of the survey with a prestamped self-addressed return envelope. Follow-up calls were made within 1 week of mailings to encourage participation. Licensed family child care providers who did not respond to the initial mailing received a second mailing and follow-up call in the final month of data collection. Those who participated in the study received a $20 gift card to a large retail store chosen for its general accessibility throughout Delaware. Data were collected during the months of October 2011 through January 2012. The study procedures were reviewed by the Nemours Institutional Review Board and received exempt status.

Data were scanned and imported from printed format into an electronic database. SPSS version 19 for Windows (IBM, Chicago, Illinois) was used for all analyses. Poststratification weighting was implemented, and the weighted percentages reflect the county distribution of the population of providers. Significance was set at .05.

### Study measures

Survey questions were designed to collect information from family child care providers on their awareness of and practices and training in nutrition, physical activity, and screen time; the characteristics of their program; and their knowledge of the physical activity regulation on moderate-to-vigorous physical activity (MVPA) pertaining to children in Delaware licensed family child care homes. Data on numerous items (eg, items on the available indoor/outdoor play space) were drawn from the Nutrition and Physical Activity Self-Assessment for Child Care (NAP SACC) instrument, an accurate measurement tool that assesses the physical activity and nutrition policies and practices in child care settings (15,16). Knowledge of the physical activity regulations was assessed via a single multiple-choice item. Family child care providers were considered to be knowledgeable if they chose the correct answer and not knowledgeable if they selected any other answer.

Compliance with the physical activity regulations was assessed via 12 multiple-choice items asking about usual practice within the family child care home. Family child care providers were asked about their compliance with the recommendation for unstructured physical activity for children older than age 1. Family child care providers were considered compliant if they answered that they provided the practice daily. Family child care providers who answered with a frequency of anything less than daily, including never practicing the regulation, were considered to be noncompliant. An item was also included that assessed family child care providers’ compliance with the structured physical activity recommendation for children older than age 2. Family child care providers were considered compliant if they answered that they provided the practice daily and noncompliant if their answer was a frequency of less than daily or never.

### Statistical analysis

Descriptive analyses were performed to provide an overview of the Delaware family child care provider population, including a description of the physical activity environment in Delaware family child care homes. Three binary logistic regression models were performed to analyze the association between family child care provider characteristics and the knowledge of and compliance with the statewide physical activity regulations and recommendations. Model 1 included the dependent variable that measured knowledge of the physical activity regulation (correct or incorrect). Independent variables measured the family child care provider’s background: 1) provider’s education, 2) number of years as a family child care provider, and 3) whether or not the provider had received formal training related to physical activity for children in the past 12 months. In addition, characteristics of the family child care home that may influence provider knowledge about the regulation were 4) affiliation with Child and Adult Care Food Program (CACFP), and 5) the percentage of children within the program receiving Purchase of Care (POC), a state program designed to make child care more affordable for low-income families.

Model 2 included the dependent variable that measured compliance with the unstructured physical activity recommendation (practiced daily or not). The independent variables included family child care provider characteristics: provider’s education, number of years as a family child care provider, and whether or not the provider had received formal training related to physical activity for children in the past 12 months. Characteristics of the family child care home that may influence regulation compliance are affiliation with CACFP; the percentage of children within the program receiving POC; the existence of family child care staff in addition to the provider participant; the availability of portable play equipment; the description of outdoor space available for children to be physically active (plenty of open running space and a track/path for wheeled toys or less than this amount of space); and the description of indoor space available for children to be physically active (for quiet play only or very limited movement, for some active play, and for all activities including running). Multicollinearity diagnostics revealed that the availability of fixed play equipment was highly correlated with the availability of portable play equipment and several other key variables, so this variable was excluded from the analysis. Because they were found to be related, the availability of portable play equipment was used as a measure of both fixed and portable play equipment. Model 3 included compliance with the structured physical activity recommendation (practiced daily or not) and all independent variables in Model 2. All independent variables were self-reported by providers via multiple choice items on the survey. The data were weighted for analysis to account for the stratified random sample design.

## Results

### Sample characteristics

Ultimately, 313 of the 663 eligible family child care providers participated in the survey (47.2% response rate). Results show that 40.3% of family child care providers reported having a high school education/general educational development diploma or less, while 36.4% reported having some college attainment, and 23.2% of providers reported having a 2-year degree or higher (Table 1). Family child care providers reported an average of 14.2 years as a child care provider. A large percentage of family child care providers (85.7%) reported having received training related to physical activity for children in the last 12 months. Nearly three-quarters of family child care providers (73.3%) were participants in the CACFP, and 38.4 of providers reported that half or more of their children were receiving POC. When asked whether they had any staff in addition to themselves, 22.8% replied yes. Nearly all participants reported having outdoor (98.9%) and indoor (99.2%) play space available. Because of the small difference within these 2 variables, they were not included in the models. When asked to describe the available outdoor play space, 54.6% described their outdoor space as having plenty of open running space and a track/path for wheeled toys. When asked to describe the available indoor play space, 23.6% reported indoor play space for all activities, including running.

**Table 1:** Characteristics of Delaware Family Child Care Programs According to Survey Participants, 2011 Delaware Child Care Provider Survey^a^

Characteristic (no. of respondents)	No. (%)^b^
**Provider location (n = 313)**	
New Castle County (outside City of Wilmington)	107 (36.8)
City of Wilmington	95 (22.5)
Kent County	53 (18.8)
Sussex County	58 (21.9)
**Education (n = 310)**	
High school diploma/GED or less	126 (40.3)
Some college	112 (36.4)
2-year degree or more	72 (23.2)
**No. of years as a child care provider (n = 312)**	
<5	45 (14.3)
5–9	64 (20.3)
10–14	54 (17.7)
15–19	63 (20.6)
20–24	46 (14.5)
≥25	40 (12.6)
**Training related to physical activity for children received in last 12 months (n = 300)**	
Yes	255 (85.7)
No	45 (14.3)
**CACFP affiliated (n = 303)**	
Yes	220 (73.3)
No	83 (26.7)
**Percentage of children receiving POC (n = 289)**	
None	107 (36.4)
<50%	70 (25.2)
≥50%	112 (38.4)
**Additional staff working in program (n = 313)**	
Yes	76 (22.8)
No	237 (77.2)
**Available outdoor play space (n = 311)**	
Yes	307 (98.9)
No	4 (1.1)
**Description of outdoor space (n = 302)**	
Less than plenty of open running space and a track/path for wheeled toys	137 (45.4)
Plenty of open running space and a track/path for wheeled toys	165 (54.6)
**Available indoor play space (n = 310)**	
Yes	307 (99.2)
No	3 (0.8)
**Description of indoor space (n = 307)**	
For quiet play only or very limited movement (eg, jumping and rolling)	76 (24.0)
For some active play (eg, jumping, rolling, and skipping)	160 (52.4)
For all activities, including running	71 (23.6)
**Availability of portable play equipment (n = 313)**	
Yes	286 (91.5)
No	27 (8.5)
**Availability of fixed play equipment (n = 313)**	
Yes	212 (69.0)
No	101 (31.0)

Abbreviation: GED, general educational development; CACFP, Child and Adult Care Food Program; POC, Purchase of Care.

a Because of missing cases within variables, numbers and percentages may not sum to sample or universe total of family child care programs in Delaware.

b Unweighted n, weighted percentages.

More than 40% of respondents knew of the regulation that each child should be provided with at least 20 minutes of MVPA for every 3 hours of care; fewer than half knew of the regulation related to the maximum length of time infants may be in confining equipment (Table 2). More than three-quarters of family child care providers reported compliance with the physical activity regulations: 86.5% of participants reported that they provide children older than 1 year at least 60 minutes of unstructured physical activity, and 78.6% reported that children older than 2 are provided at least 60 minutes of structured physical activity. In a typical week, 53.7% of respondents reported that children older than 1 are never sedentary for more than 60 minutes daily during waking hours. More than half of family child care providers (53.2%) reported that they always actively play with children (length of time associated with this practice is unknown).

**Table 2:** Knowledge and Practice of Physical Activity Regulations, 2011 Delaware Child Care Provider Survey (n = 313)^a^^,^ b

Regulation (no. of respondents)	Response
**Knowledge of physical activity regulations^c^**	**Correct, No. (%)**
Each child should be provided with at least 20 min of moderate-to-vigorous-physical activity for every 3 h of care (n = 312).	131 (41.9)
Infants may be in confining equipment for no more than 30 min during waking hours (n = 311).	141 (45.6)
**Practice of physical activity regulations^d^**	**Practiced Daily, No. (%)**
During a typical week, children aged >1 provided at least 60 total minutes of unstructured physical activity (n = 302).	258 (86.5)
During a typical week, children over the age of 2 provided at least 60 total minutes of structured physical activity (n = 297).	229 (78.6)
During a typical week, children over the age of 1 outdoors (n = 288).	210 (73.5)
**Practice of physical activity regulations^d^**	**Practiced Never, No. (%)**
During a typical week, children over the age of 1 sedentary for more than 60 min during waking hours (n = 255).	136 (53.7)
**Staff behavior related to physical activity for children^d^**	**Practiced Always, No. (%)**
Never restricting physical activity as punishment (n = 296).	244 (82.8)
Never using physical activity as a form of punishment (n = 296).	285 (96.6)
Provider actively plays with children (n = 296).	157 (53.2)
Provide opportunities for physical activity indoors when outdoor play is not possible because of weather (n = 302).	212 (71.5)

a Because of missing cases within variables, numbers and percentages may not sum to sample or universe total of family child care programs in Delaware.

b Unweighted n, weighted percentages.

c Items have been recoded from categorical into indicator variables. Original survey answer categories were “10 minutes,” “20 minutes,” “30 minutes,” and “not sure.”

d Items have been recoded from categorical into indicator variables. Original survey answer categories were “never,” “1–2 times per week,” “3–4 times per week,” and “daily.”

e Items have been recoded from categorical into indicator variables. Original survey answer categories were “never,” “some of the time,” “most of the time,” and “always.”

### Model 1. Knowledge of the moderate-to-vigorous physical activity regulation

When controlling for the covariates, education had an independent significant contribution to a family child care provider’s knowledge of the regulation of 20 minutes of MVPA for every 3 hours of care (Wald *F* = 4.59, *P* = .01) (Table 3). Family child care providers who had a 2-year college degree or more were 2.27 times as likely to know the physical activity regulation (95% confidence interval [CI], 1.16–4.43) as providers who had a high school education or a general educational development diploma or less.

**Table 3:** Family Child Care Providers’ Self-Reported Physical Activity Knowledge and Practice, 2011 Delaware Child Care Provider Survey (n = 313, N = 1,037)

Characteristic	Model 1^a^ (n = 894), OR (95% CI)	Model 2^b^ (n = 843), OR (95% CI)	Model 3^c^ (n = 830), OR (95% CI)
**Education**			
High school diploma/GED or less	1 [Referent]		
Some college	0.80 (0.43–1.48)	0.945 (0.38–2.33)	1.24 (0.54–2.83)
2-year college degree or more	2.27 (1.16–4.43)^d^	1.69 (0.58–4.94)	0.67 (0.28–1.58)
**No. of years as a child care provider**			
<5	1 [Referent]		
5–9	0.66 (0.28–1.59)	1.28 (0.29–5.70)	1.16 (0.32–4.17)
10–14	0.54 (0.22–1.33)	1.38 (0.32–5.99)	0.76 (0.23–2.49)
15–19	0.31 (0.12–0.79)	1.17 (0.29–4.71)	1.44 (0.45–4.68)
20–24	1.05 (0.42–2.65)	1.08 (0.24–4.86)	2.29 (0.55–9.52)
≥25	0.80 (0.31–2.05)	1.37 (0.27–6.86)	0.76 (0.21–2.77)
**Participant received formal training related to physical activity for children in past 12 months**			
Yes	1 [Referent]		
No	0.86 (0.41–1.82)	1.14 (0.37–3.56)	0.55 (0.21–1.46)
**CACFP-affiliated**			
Yes	1 [Referent]		
No	1.13 (0.59–2.17)	0.84 (0.33–2.17)	0.66 (0.31–1.45)
**Percentage of children receiving Purchase of Care**			
None	1 [Referent]		
<50	0.94 (0.48–1.82)	0.36 (0.14–0.91)	0.52 (0.22–1.22)
≥50	0.66 (0.35–1.22)	0.85 (0.35–2.11)	0.88 (0.39–1.99)
**Additional staff working in program**			
Yes	1 [Referent]		
No	NA	1.17 (0.48–2.85)	0.75 (0.32–1.75)
**Availability of portable equipment**			
Not available	1 [Referent]		
Available	NA	2.49 (0.77–8.13)	2.52 (0.91–6.96)
**Outdoor space**			
Does not have plenty of open running space and a track/path for wheeled toys	1 [Referent]		
Has plenty of open running space and a track/path for wheeled toys	NA	2.4 (1.05–5.50)^e^	1.08 (0.52–2.27)
**Indoor space**			
For quiet play only/for very limited movement (eg, jumping and rolling)	1 [Referent]		
For some active play (eg, jumping, rolling, and skipping)	NA	1.97 (0.88–4.42)	1.35 (0.67–2.72)
For all activities, including running	NA	3.43 (0.99–11.83)	11.20 (2.41–52.18)^d^

Abbreviations: GED, general educational development; MVPA, moderate-to-vigorous physical activity; OR, odds ratio; CI, confidence interval; CACFP, Child and Adult Care Food Program; NA, not applicable.

a Model 1 is knowledge of the regulation that children must be provided with 20 min of moderate to vigorous physical activity for every 3 h of care. The reference category is “incorrect”; “n” and confidence intervals are weighted.

b Model 2 is compliance with the recommendation that children older than 1 year are provided with 60 min of unstructured activity daily. The reference category is “provided less than daily or never”; “n” and confidence intervals are weighted.

c Model 3 is compliance with the recommendation that children older than 2 years are provided with 60 min of structured activity daily. The reference category is “provided less than daily or never”; “n” and confidence intervals are weighted.

d Significant at *P* < .001.

e Significant at *P* = .05.

### Model 2. Compliance with the unstructured physical activity recommendation

When controlling for the covariates, the description of the available outdoor play space had an independent and significant association with a family child care provider’s compliance with the unstructured physical activity recommendation (Wald *F* = 4.37, *P* = .04). Family child care providers who reported having plenty of open running space and a track/path for wheeled toys were 2.4 times as likely to report providing at least 60 minutes daily of unstructured physical activity (95% CI, 1.05–5.50) as those who did not report having this much available outdoor play space.

### Model 3. Compliance with the structured physical activity recommendation

With all other covariates controlled for, the description of the physical space available inside had an independent and significant contribution to family child care provider’s compliance with the structured physical activity recommendation (Wald *F* = 4.80, *P* = .009). This was the only significant predictor in this model. Family child care providers who described their available indoor space as room for all activities, including running, were 11.2 times as likely as providers who described their available indoor space as one for quiet play only or for limited movement (eg, jumping and rolling) to report following the recommendation for the daily practice of the structured physical activity.

## Discussion

This study of the family child care provider environment, knowledge and practice of physical activity regulations and recommendations allowed us to analyze the association between family child care provider characteristics and knowledge of and compliance with physical activity regulations and recommendations. Our results suggest that fewer than half of family child care providers know of the physical activity regulations for MVPA and the regulation on length of time infants may be in confining equipment while awake. Advocates of the recommendations should continue to train and educate all family child care providers, with the goal of making all providers aware of the level of MVPA to be provided to children in their care. Not surprisingly, we found that education was a significant predictor of family child care provider knowledge of the physical activity regulation for MVPA for children older than 2. Future researchers may want to consider and include other covariates that may explain more clearly patterns among these providers.

Our results suggest that available physical space within the family child care environment is an important factor that affects the provision of physical activity to children within family child care programs. In fact, we found that the type of space available for both indoor and outdoor areas had an effect on reported physical activity. Existing research supports the finding that play space has a positive influence on children’s level of physical activity (10). Our results suggest that adequate indoor play space, specifically enough room for all activities including running compared with only enough room for quiet play or limited movement, increases the likelihood that the recommendation on structured physical activity is practiced daily within family child care environments. The available physical space is an important area of focus for advocates of physical activity recommendations. Practitioners who train and educate providers to implement physical activity regulations should consider the effect of both indoor and outdoor space on structured and unstructured physical activity, while working within the existing physical space constraints of family child care provider programs. They should provide ideas and examples of structured and unstructured physical activity practices that can work in various play space settings. There may also be opportunities to link family child care providers to public play spaces (eg, schools, parks) especially in urban areas where individual family child care providers may have limited outdoor space. These opportunities were not fully explored in our data set because of the relatively small amount of urban area in the state.

Several factors may influence the interpretation of the results of this study. Despite our best efforts, our response rate was only 47.2%, limiting our ability to make inferences about nonresponding family child care providers. To explore nonresponse bias, we compared the demographics of our respondents with those in an existing database from another survey on statewide nutrition and physical activity training called Team Nutrition conducted in 2011 with all CACFP participants in Delaware. The results of this exploration suggest that our respondents were slightly more educated than the overall population of family child care providers in the state. In addition, family child care providers within the city of Wilmington were underrepresented in our sample. Although sampling weight did control for this factor, it should be taken into consideration when attempting to generalize. In addition, considering the sampling methodology, although this sample may be a reasonable representation of family child care providers within the state of Delaware, this study cannot be generalized to providers in other US states or other types of care settings.

Because of length limitations, this survey assessing Delaware family child care environments did not include an exhaustive list of items on physical activity-related training, measuring only family child care provider’s training within the past year. It did not ask about past provider training experience or the quality of training. Therefore, this concept of provider training was excluded from the analysis. Future studies and analyses focusing on the family child care setting should include type, frequency, and duration of training received. Furthermore, future research should include triangulation of data methods to include both observed and self-reported measures of the frequency and intensity of children’s physical activity in the family child care environment. The extent to which respondents were intentionally or unintentionally inaccurate about the characteristics of their site, background, or practices is unknown. The validity of our self-reported data could be increased by adding observational measures; given resource limitations, we were unable to add these measures. However, future researchers may want to consider validating certain self-reported measures with observation.

This study provides important insight into the Delaware family child care environment, filling a gap in the existing literature. Public health policy makers and practitioners focusing on children’s physical activity should consider not only the availability of the outdoor and indoor physical space but also the description of that space necessary for structured and unstructured physical activities.
